# Lung Ultrasound: The Cardiologists' New Friend

**DOI:** 10.5935/abc.20170169

**Published:** 2017-12

**Authors:** Marcelo Haertel Miglioranza, Antonio Carlos Sobral Sousa, Caroline de Souza Costa Araujo, Marcos Antonio Almeida-Santos, Luna Gargani

**Affiliations:** 1 Instituto de Cardiologia do Rio Grande do Sul - Fundação Universitária de Cardiologia, Porto Alegre, RS; 2 Núcleo de Pós-graduação em Ciências da Saúde da Universidade Federal de Sergipe, São Cristóvão, SE; 3 Núcleo de Pós-Graduação em Saúde e Ambiente da Universidade Tiradentes, Aracaju, SE - Brazil; 4 Instituto de Fisiologia Clínica - Conselho Nacional de Pesquisa de Pisa - Italy

**Keywords:** Ultrasonography, Heart Failure, Pulmonary Edema, Diagnosis, Differential

About 200 years ago, French physician Theophile Hyacinthe Laënnec (1781-1826) invented the stethoscope (from the Greek *stethos* = thorax, and *skopein* = to explore). Initially, the medical community was skeptical of the usefulness of the stethoscope and there was initial resistance to its use: <that it would be widely used, despite its value, is extremely doubtful, as its beneficial application takes a lot of time and causes many problems for both the patient and the physician>. However, in a short period of time, the stethoscope became a key component of the physical examination, and auscultation gained an outstanding value, promoting great advances in the diagnosis and management of patients with heart and lung diseases.^1^ Given the importance of this instrument, the stethoscope became iconic, constituting a symbol of the knowledge of the Hippocratic art - it is difficult to recognize another symbol that identifies the physician as strongly as a stethoscope adorning the neck of its user.

Several decades have passed, and now we are faced with a similar scenario: another paradigm to be changed. Over a long period, the scientific community believed that the lungs would be outside the scope of ultrasonic investigation: <because ultrasound energy dissipates rapidly into the air, ultrasonographic imaging is not useful for the assessment of lung parenchyma>. This statement is true under normal physiological conditions. However, the occurrence of water in the pulmonary structure creates an acoustic window that allows the echocardiographer to identify the presence of congestion, as well as to perform a semiquantitative analysis on it. Point-of-care ultrasonography (centered examination, that is, performed at the patient’s own care site, often by the physician/care provider) emerged as an extension of the physical examination, and lung ultrasonography was proposed as part of it to detect and to estimate interstitial pulmonary edema. Therefore, cardiologists may now have this ultrasound technology as part of the clinical examination, which can be applied both at the bedside and in the office, and proposes to answer specific questions in a decision-making approach.

## The role of pulmonary congestion in heart failure and the limits of the traditional clinical examination

Pulmonary congestion, such as low cardiac output, is a preponderant element in patients with heart failure (HF), which is considered an important cause of hospital admissions and death.^2,3^ Thus, the identification of pulmonary extravascular fluid in patients with HF can be used as an aid in strategies to optimize clinical therapy.

Traditionally, assessment of pulmonary congestion has been based on the patient’s clinical status and physical examination. However, this evaluation presents limitations even for skilled professionals, showing high specificity, but low sensitivity for the detection of pulmonary congestion.^4,5^ Thus, cases of decompensation are often recognized at a very late stage of clinical congestion, so frequent hospitalizations are not avoided. In the cascade of congestion, the clinical manifestation represents a final stage, different from hemodynamic congestion (increase in left ventricular filling pressure), which is pulmonary and systemic.^6^ Pulmonary congestion corresponds specifically to the presence of extravascular pulmonary fluid, which can be evaluated by lung ultrasound.

## The added value of lung ultrasonography

Lung ultrasound has emerged as an additional assessment to the tests and strategies already used in the clinical setting. However, many studies have shown that this test has comparable results to traditional complementary methods, and therefore can be used as a substitute. In fact, it is difficult to claim the full applicability and “sufficiency” of a single complementary method alone. An example of this is the restriction of the use of radiological examination during management and the difficulty in bearing the costs of the BNP (brain natriuretic peptide) dosage. However, lung echocardiography, considering that the echocardiograph is already available in a given institution, becomes a plausible alternative to be used alone or in face of the restrictions pointed out for radiographic examinations and sophisticated biochemical measurements.

**Figure 1:**
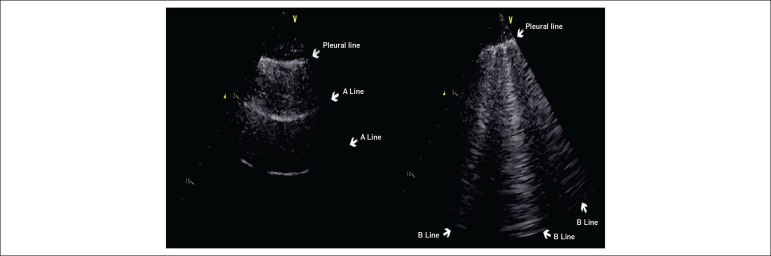
Lung ultrasound images showing a normal lung and a lung with signs of congestion. On the right, we see an aerated lung and the only structure that can be identified is the pleura, appearing in the image as a horizontal hyperechogenic line. From the pleural line, we see several horizontal lines at regular intervals (A lines). On the left, we see a lung with interstitial edema; the acoustic discrepancy between the air and the surrounding tissues changes, generating a vertical reverberation artifact (B lines).

Detection of B lines (previously referred to as lung comets) through lung ultrasound has been proposed as a simple, noninvasive and semiquantitative tool to evaluate the presence of extravascular pulmonary fluid.^7,8^ When the lung is normally aerated, no B line is visible and the image is “black”. On the other hand, when the pulmonary vessels become engorged and the fluid transpires into the interstitium, the B lines begin to appear and the image becomes “black and white”. With alveolar edema, the image is completely “white”, full of B lines (Figure 1). This signal was initially proposed for the differential diagnosis of acute dyspnea, it is now part of the recommendations of the European Society of Cardiology for the pre- and in-hospital management of acute HF,^9^ and is also part of the recommendations of the European Association of Cardiovascular Imaging and of the Acute Cardiovascular Care Association on the use of echocardiography in intensive cardiovascular care^10^ and emergency care.^11^ Several studies have demonstrated the relationship between B lines and pulmonary extravascular fluid, pulmonary capillary filling pressure,^12^ NT-proBNP^13^ and E/e' ratio in patients with HF.

Lung ultrasound can also identify clinically silent pulmonary edema^14-16^ and is an independent predictor of events in patients with acute HF,^17,18^ chronic HF,^19,20^ acute coronary syndromes,^21^ hemodialysis^22,23^ or acute dyspnea and/or chest pain,^24^ suggesting its additional value for improving the hemodynamic profile and optimizing treatment.

The sensitivity and specificity of pulmonary echocardiography for the detection of B lines have ranged from 85 to 98%, and from 83 to 93%, respectively.^14,25^

## Advantages and limitations

The implantation of lung ultrasound requires a learning curve, as it usually occurs in several complementary exams. On the other hand, implantation is highly accessible, and can be performed from basic ultrasound technology, including pocket devices. It is a fast, inexpensive, non-invasive and radiation-free procedure that allows for use in stable and unstable patients, as well as simultaneously to physical examination, and in resuscitation and hemodynamic stabilization.

However, to avoid erroneous interpretations of B lines, the key is to contextualize with the clinical status, as this sign does not necessarily imply a cardiogenic etiology.^26,27^ When the presence or persistence of B lines does not show a correlation with the clinical status of HF, other diagnostic possibilities, such as pulmonary fibrosis in users of amiodarone, non-cardiogenic pulmonary edema or interstitial lung disease, should be considered.^28^

In addition, lung ultrasonography may contribute to the development of new prognostic scores in patients with heart failure, since pulmonary congestion is one of the main predictors of fatal events in this group of individuals.^29^

## Conclusion

The use of lung ultrasonography is therefore promising as a complementary method in cardiology. In this article, the main arguments for its use in everyday clinical practice were presented. Just as the introduction of the stethoscope has ushered in a new era in clinical diagnosis, we believe that the incorporation of point-of-care ultrasound has enough potential to expand the boundaries of traditional physical examination and, through a new praxis, broaden the physician’s senses.
